# Necroptosis Plays a Crucial Role in Vascular Injury during DVT and Is Enhanced by IL-17B

**DOI:** 10.1155/2022/6909764

**Published:** 2022-08-19

**Authors:** Yunyan Li, Jianfu Chen, Yuan Yang, Yuxue Wang, Yong Zhang, Yan Zhou, Yan Bao, Zongmei Zhang, Yongping Lu

**Affiliations:** ^1^Department of Ultrasound, The Affiliated Hospital of Yunnan University (The Forth Affiliated Hospital of Kunming Medical University, The Second People's Hospital of Yunnan Province), Kunming, Yunnan, China; ^2^Department of Vascular Surgery, The Affiliated Hospital of Yunnan University (The Forth Affiliated Hospital of Kunming Medical University, The Second People's Hospital of Yunnan Province), Kunming, Yunnan, China; ^3^Department of Pathology, The Third Affiliated Hospital of Kunming Medical University, Yunnan Tumor Hospital, Yunnan Cancer Center, Kunming, Yunnan, China

## Abstract

**Background:**

This study investigated whether vascular endothelial necroptosis is involved in deep vein thrombosis (DVT) and how IL-17B facilitates necroptosis signaling.

**Methods:**

The DVT mouse model was induced by ligation of the IVC. The cross-sectional area of thrombus increases and the thrombus occupied the entire venous lumen at 48 h after ligation. Meanwhile, the increased expression of p-RIP3/RIP3 was most pronounced at 48 h after ligation, and the p-MLKL/MLKL peaked at 72 h.

**Results:**

Based on Illumina sequencing and KEGG pathway analyses, the activated RIP3/MLKL is associated with increased IL-17B. With thrombus formation, IL-17B was upregulated and enhanced the expression of RIP3 and MLKL in the IVC wall, as well as their phosphorylation levels (all *P* < 0.05, the comparison group consisted of the control group, DVT group, DVT/IL-17B group, and DVT/anti-IL-17B group). The p-RIP3/RIP3 and p-MLKL/MLKL ratios were reduced by anti-IL-17B. Similarly, the weight and cross-sectional area of the thrombi were increased by IL-17B and decreased by the IL-17B antibody. IL-17B had a smaller effect on thrombosis in knockout mice compared with WT mice. In vitro, the IL-17B protein expression and the level of RIP3 and MLKL phosphorylation increased high in the OGD cells, accompanied by increased expression of IL-6 and TNF-*α*. IL-17B enhanced the expression of IL-6 and TNF-*α* but had little effect on the IL-6 and TNF-*α* after transfected with siRIP3 or siMLKL. Similarly, the plasma IL-17B, IL-6, and TNF-*α* were significantly increased after thrombosis in WT mice, and enhanced by IL-17B. But IL-17B did not increase the plasma IL-6 and TNF-*α* in knockout mice.

**Conclusions:**

In conclusion, those results suggest that vascular endothelial necroptosis plays a crucial role in vascular injury and IL-17B could enhance the necroptosis pathway.

## 1. Introduction

Deep vein thrombosis (DVT) and pulmonary embolism (PE) are called venous thromboembolism (VTE), which is one of the main disease burdens in the world [1]. At least 1 in 12 adults over the age of 45 will develop VTE during their lifetime, and few people know about VTE [2]. Approximately half of VTE events occur unpredictably, and the risk of VTE is not eliminated even when appropriate thromboprophylaxis is carried out [3]. Patients with VTE have a long-term increased risk of dying, in particular, mortality risk peaks within 30 days onset that for patients with PE is 31% and 3% of those with DVT [4]. Effective anticoagulation can reduce the incidence of fatal PE by 50% [5], and systemic thrombolytic therapy can reduce mortality in patients with massive acute PE [6]. However, over 50% of patients may suffer postthrombotic syndrome (PTS) and nearly 30% of patients may develop recurrent thromboembolism in long term even with appropriate anticoagulant therapy [7], which seriously affect the patients' quality of life and even threaten their lives.

Blood stasis, hypercoagulability, and endothelial injury have been considered to be traditional triggers of venous thrombosis [8]. Among them, the role of endothelial injury is becoming more apparent, but significant vascular wall injury does not seem to be a necessary or sufficient condition for thrombosis. Experimental venous thrombosis does not result from damage to the endothelium caused by squeezing the vein. However, a persistent thrombus can aggravate the injury to the vascular endothelium, both physically with distension and from inflammation [9], which is associated with re-thrombosis [10, 11]. Recurrent ipsilateral DVT and residual venous thrombosis (RVT) are important risk factors for PTS [12, 13]. Removing the luminal thrombus rapidly will restore blood flow and limit the damage to the vein wall, and ultimately reduce PTS [14]. But recovery of blood flow (RBF) after 4 days of thrombosis has little benefit for vein wall fibrosis [15]. In addition, the acute phase of venous thrombosis (VT) impairs the endothelial function of the vein wall while preserving the function of vascular smooth muscle cells, which may be the mechanism promoting PTS [16]. Therefore, we hypothesize that the vascular endothelium is damaged in the early stage of thrombosis, and if the damage is irreversible, even anticoagulation and thrombolytic therapy to restore blood flow will have no effect on the occurrence of PTS. But the mechanism of vascular wall damage after thrombosis is often neglected.

At present, the mechanism of vascular endothelial injury in VT mainly involves apoptosis, and the control of vascular endothelial cell apoptosis can reduce thrombosis [17, 18]. The process of apoptosis has been shown to have no release of cell contents and therefore no inflammatory response. Moreover, when apoptosis occurs in inflammatory lesions, the inflammatory response may be terminated [19]. In recent years, more and more studies have found that inflammatory response is an important factor in vascular wall injury [20]. Therefore, research on cell necrosis is a necessary way to investigate the relationship between DVT and inflammation. Necroptosis is a novel cell death type distinct from apoptosis and necrosis, which has been demonstrated to be involved in endothelial dysfunction [21, 22]. The receptor-interacting protein kinase 3 (RIP3), along with the mixed lineage kinase domain-like pseudokinase (MLKL), is the main elements of the necroptosis pathway [19, 23, 24]. The activation of RIP3 plays a key role in determining whether cell apoptosis or necrosis occurs [25]. Activated RIP3 inhibits apoptosis and then initiates necroptosis, and inactivated RIP3 triggers caspase-8-mediated apoptosis through RIP1 [25]. As the terminal protein in the program of necroptotic cell death process [26], MLKL has only been credibly attributed functions in necroptosis signaling, and the main role of MLKL activation is its phosphorylation by RIPK3 [27–29]. RIP3 and MLKL are specifically phosphorylated to perform the function of causing necrosis when necroptosis occurs, and their phosphorylated proteins can be used as specific markers of necroptosis. Nevertheless, whether necroptosis is involved in endothelial injury during thrombosis remains unknown.

In previous study, we found that the phosphorylation levels of RIP3 and MLKL were significantly increased in vascular wall during acute DVT. To understand the possible mechanisms of necroptosis-mediated endothelial injury during thrombosis, the IVC tissues of DVT RIP3 knockout mice, MLKL knockout mice, and WT mice were sequenced using the Illumina sequencing platform to find differential genes. And the Kyoto Encyclopedia of Genes and Genomes (KEGG) enrichment analyses indicated that the differentially expressed interleukin 17B (IL-17B) in IVC tissues during thrombosis was significantly associated with the RIP3/MLKL signaling pathway, but the regulation effect o thef IL-17B RIP3/MLKL pathway in acute DVT remains unknown.

IL-17B was discovered in 2000 and is a less well-characterized cytokine among the IL-17 family [30]. Although many studies on the IL-17 family have been carried out in recent years, most scholars have focused on the IL-17A subtype [31]. IL-17B may elicit effects similar to those of IL-17A in target cells, but its functions may also be different (sometimes opposite) from those of IL-17A in specific tissues [32]. IL-17A was shown to play an important role in the development of endothelial dysfunction [33], and it was found to promote DVT formation by activating endothelial cells and promoting platelet aggregation and neutrophil infiltration [34], but IL-17B was not studied well in DVT.

Therefore, the aim of this study was to examine the effects of RIP3, MLKL, and IL-17B on cell viability in vitro and to examine the role of IL-17B in regulating RIP3 and MLKL in the vascular wall, thus modulating thrombosis, in a DVT mouse model. The results could provide novel insights into the mechanisms of DVT, helping prevent recurrences and complications.

## 2. Materials and Methods

### 2.1. In Vivo Study

All animal experiments were approved by the animal experimental ethical committee of Kunming Medical (no.: KMMU 2019054)) University. Male and female adult wild-type (WT) mice, RIP3-knockout (RIP3-/-) mice, and MLKL-knockout (MLK-/-) mice were purchased from Leike Jingda Animal Model Technology Company (Hunan, China). A venous thrombosis model was induced by ligation of the inferior vena cava (IVC) [35]. The mice were anesthetized with 1% pentobarbital sodium (1 ml/kg). The abdominal wall was disinfected three times with 3% iodine. A median abdominal incision was made. The IVC was isolated with ophthalmic tweezers under a stereoscope. A 3-0 nylon suture was placed parallel to the surface of the IVC, both the IVC and the 3-0 nylon suture was tied around by a 6-0 nylon suture at the bifurcation of the left renal vein. The 3-0 nylon suture was then removed. All branches were ligated. The incision was closed by a two-layered suture. The control animals had the same procedure but without IVC ligation.

In order to understand the formation regularity of DVT and the trends of RIP3/MLKL signals during DVT, WT mice were divided according to different stenosis time (control, 2 h, 6 h, 12 h, 24 h, 48 h, and 7 days), with six mice in each group. A total of fifty-two mice were used, and five mice died because of bleeding. The cross-sectional thrombus area was measured on HE images using Image-Pro Plus 6.0. The RIP3 and MLKL protein expressed levels and their phosphorylation levels in the IVC tissues were detected by Western blot. The RIP3 mRNA and MLKL mRNA of IVC tissue were qualified by quantitative real-time polymerase chain reaction.

WT mice were divided into four groups to evaluate the effects of IL-17B on RIP3 and MLKL: DVT group (*n* = 18, IVC stenosis), DVT/IL-17B group (*n* = 18, IVC stenosis and intravenous injection with 50 *μ*g/kg IL-17B through the jugular vein approximately 30 min prior to IVC ligation [34]), DVT/anti-IL-17B group (*n* = 18, IVC stenosis and intravenous injection with 50 *μ*g/kg IL-17B antibody through the jugular vein approximately 30 min prior to IVC ligation), and control group (*n* = 18, no treatment). Six mice in each group were used for ELISA analysis, six mice were used for immunohistochemistry, and six mice were used for western blot analysis. For this step, seventy-seven WT mice were used, but five mice died because of bleeding caused by a surgical error.

To further investigate the effects of IL-17B on RIP3 and MLKL of IVC tissues, we conducted the following six animal experiments: WT mice, RIP3-/- mice, and MLKL-/-mice with IVC stenosis with or without IL-17B treatment (*n* = 18 each group). A total of 118 mice (WT mice (*n* = 37), MLKL-/- mice (*n* = 40), and RIP3-/- mice (*n* = 41)) were used. Ten mice (8.5%) died (two because of overdose and eight because of bleeding). Mice that bled and died before IVC tissue obtained time were not included in the analysis.

### 2.2. Histological Examination of the Thrombus

At different time points after IVC ligation (2 h, 6 h, 12 h, 24 h, 48 h, and 7 days), the mice were euthanized by CO_2_ asphyxiation. The IVC tissues (including the thrombus) were obtained from just below the left renal vein to the iliac vein bifurcation, and the weight was measured on an electronic balance. IVC tissues from the control group were used for comparison. Sections of the specimens 3 mm below the IVC ligation were fixed with 4% paraformaldehyde for hematoxylin and eosin (HE) analysis, and the cross-sectional thrombus area was measured using Image-Pro Plus 6.0 (Media Cybernetics, Inc., Rockville, MD, USA).

### 2.3. Illumina Sequencing

Based on the Illumina sequencing platform (Annoroad Gene Technology Corporation, Beijing, China), the IVC tissues of DVT RIP3-/- mice, MLKL-/- mice, and WT mice (induced by ligation of the IVC for 48 h) were sequenced. Total RNA was extracted from IVC tissues, the quality was assessed, and the library was constructed. An Illumina platform (Illumina, Inc., San Diego, CA, USA) was used to sequence the library, and the sequencing strategy was PE150. Genes with ∣log2 fold change | ≥1 and *q* < 0.05 were considered to be significantly different. The upregulated and downregulated genes were compared, differentially expressed heat maps were drawn, and protein interaction network analysis and KEGG enrichment analysis were performed to identify the pathways with significantly different enrichment.

### 2.4. Immunohistochemistry

IVC tissues containing thrombus were fixed on slides and rinsed with PBS. The slides were blocked in 5% BSA in PBS for 1 h and treated with the primary antibodies at 4°C overnight, namely, rabbit anti-MLKL (p-S345) antibody (1 : 1000; #ab196436; Abcam, Cambridge, United Kingdom), rabbit anti-RIP3 (p-S232) antibody (1 : 50; #ab195117; Abcam, Cambridge, United Kingdom), and rabbit anti-IL-17B antibody (1 : 100; #ab79056; Abcam, Cambridge, United Kingdom). The slides were incubated at 37°C for 40 min with the secondary antibody (goat anti-rabbit IgG antibody, Abcam, Cambridge, United Kingdom) after washing with PBS three times and stained with hematoxylin for 5 min. The images were revealed using HRP-DAB and observed using a Lab.A1 microscope at 200x (Carl Zeiss GmbH, Oberkochen, Germany). The positive expression of the protein was observed by an optical microscope and showed brownish-yellow particles. Five high-power fields were randomly selected for observation using the double-blind method. The Image-Pro Plus 6.0 software (Media Cybernetics, Inc., Rockville, MD, USA) was used to calculate the positive rate.

### 2.5. Expression of RIP3 and MLKL

From the specimens prepared above, the vein wall was separated from the thrombus. The RIP3 and MLKL protein expression and their phosphorylation levels in the IVC wall were quantified by Western blot. The expression of IL-17B in the IVC tissue was detected by ELISA. The mRNA expression of MLKL and RIP3 in the IVC wall was evaluated by quantitative real-time PCR.

### 2.6. Cell Culture

Human umbilical vein endothelial cells (ECV304) were a gift from the China Center for Type Culture Collection and maintained in DMEM supplemented with 10% fetal bovine serum (Bioind, Kibbuiz, Israel) in 5% CO_2_ at 37°C.

### 2.7. Oxygen-Glucose Deprivation (OGD) Cell Model

Venous blood is the only source of oxygen supply to the venous wall. The decrease of new blood supply after thrombosis leads to local hypoxia of the venous wall, which links blood flow stagnation with subsequent damage to the vascular wall. Therefore, we established a model of vascular endothelial injury after thrombosis by means of OGD. The cell line uses ECV304 for testing. ECV304 cells were cultured in hypoxia culture medium without sugar. The expression of IL-17B protein was extracted for 2 h, 4 h, 6 h, 12 h, and 24 h after constructing the OGD cell model. After the OGD model was established, IL-17B (200 ng/ml) was added for 24 h intervention, and then, the protein was extracted.

### 2.8. Transfection

The lentivirus vector system was purchased from Guangzhou Funeng Co. (Guangzhou, China). The lentiviral vectors expressed small interfering RNA (siRNA) targeting RIP3 or MLKL. The sequence used for the Sema4D siRNA was from a previous report [36]. Primer sequences were determined with the siCatch optimized siRNA design algorithm (MLKL siRNA sequence: 5′-CAG TGC CGG CGC CTG GGC CAC CG-3′; RIP3 siRNA sequence: 5′-GTT CTC CCC TGT GTA TTC TGA CG-3′). The lentiviruses were prepared and used as previously reported. The ECV304 cells were cultured to a confluence of 70% and subjected to instantaneous transfection with siRNA at a final concentration of 20 nM with the Lipofectamine 2000 transfection reagent (Invitrogen Inc., Carlsbad, CA, USA), and the medium was changed after 6 h of transfection. Transfection lasted for 48 h, the efficiency of MLKL and RIP3 interference was detected by Western blot. IL-17B (0.05 *μ*g, final concentration of 200 ng/ml) was added at 48 h after transfection. The inflammatory cytokines IL-6 and TNF-*α* in each group were detected by ELISA.

### 2.9. Western Blot

The RIP3 and MLKL protein expressed levels and their phosphorylation levels in the IVC tissues were detected by Western blot. Proteins were extracted with RIPA lysis buffer (Elbscience Institute of Biotechnology, Wuhan, China) containing a protease inhibitor cocktail (Millipore Corp., Billerica, MA, USA). Protein concentrations were detected using a bicinchoninic acid protein assay kit (Buijia Biotechnology, Xiamen, China). The protein samples (50 *μ*g) were loaded onto gels for sodium dodecyl sulfate-polyacrylamide gel electrophoresis, and proteins were transferred onto PVDF membranes (Millipore Corp., Billerica, MA, USA). *β*-Actin was used as a loading control. The membranes were blocked with 5% skim milk at 25°C for 40 min and separately incubated at 4°C for 24 h with various primary antibodies, including mouse anti-MLKL antibody (1 : 1000; #ab243142; Abcam, Cambridge, United Kingdom), rabbit anti-MLKL (p-S345) antibody (1 : 2000; #ab196436; Abcam, Cambridge, United Kingdom), rabbit anti-RIP3 (p-S232) antibody (1 : 1000; #ab195117; Abcam, Cambridge, United Kingdom), rabbit anti-cleaved caspase-3 antibody (1 : 3000; #ab2302; Abcam, Cambridge, United Kingdom), and mouse anti-*β*-actin antibody (1 : 5000; #ab6276; Abcam, Cambridge, United Kingdom). Afterward, the membranes were washed in PBS three times and incubated with the secondary antibody (goat anti-rabbit IgG; 1 : 5000; #ab205718; Abcam, Cambridge, United Kingdom). The protein membranes were treated with Super Signal reagents (Thermo Fisher Scientific, Waltham, MA, USA) for visualization. The protein bands were imaged using an ImageQuant QuickChemi 5100 system (Monad Biotech Co., Ltd., Shanghai, China). The grayscale analysis was done by ImageJ software (version 1.46; National Institutes of Health, Bethesda, MD, USA), and the relative protein expression was calculated.

### 2.10. Quantitative Real-Time Polymerase Chain Reaction (Q-PCR)

Total RNA was prepared using TRIzol (Invitrogen Inc., Carlsbad, CA, USA). RNA samples were used to synthesize cDNA with a QuantiTect Reverse Transcription kit (Takara Bio, Otsu, Japan) under the following reaction conditions: 42°C for 60 min and 70°C for 5 min. Q-PCR was carried out with SYBR® Premix Ex TaqTM II (GeneCopoeia, Rockville, MD, USA) on an Applied 7000 Real-Time PCR System (Applied Biosystems, Foster City, CA, USA), according to the manufacturer's instruction. The following primers were used: GAPDH (forward: 5′-CTT TGG CAT TGT GGA AGG GCT C-3′, reverse: 5′-GCA GGG ATG ATG TTC TGG GCA G-3′), MLKL (forward: 5′-ATC TTG CGT ATA TTT GGG ATT TG-3′, reverse: 5′-TCT GCT TTA GTG CTC TTT GCT GT-3′), and RIP3 (forward: 5′-CCA GAG AGC CAA GCC AAA GAG-3′, reverse: 5′-CAG CCA CGG GGT CAG AAG ATG-3′). The housekeeping gene GAPDH was used for normalization. The mRNA quantification was determined using the formula 2^-*ΔΔ*Ct^ method after normalization to GAPDH [37].

### 2.11. Enzyme-Linked Immunosorbent Assay (ELISA)

IL-17B expression levels in the IVC tissues were detected using an ELISA kit (JL31840-96 T Jianglai Biological Technology, Shanghai, China), according to the manufacturer's instructions. The sensitivity of IL-17B detection was 0.5 ng/ml. All samples were analyzed in triplicates.

### 2.12. Sirius Red Staining and Analysis

After routine dewaxing of paraffin sections, the sections were placed in lapis lazis blue solution for 5-10 min, washed with distilled water for 3 times, and stained with Sirius red saturated picric acid for 15-20 min. Anhydrous alcohol directly differentiates and dehydrates, xylene is used to transparent and neutral gum to sealed. The staining results showed that the collagen was red, the nucleus was green, and the others were yellow. Observation with lab.A1 microscope (200x). Five visual fields were randomly selected for double-blind observation. Collagen content was calculated using Image-Pro Plus 6.0 software.

### 2.13. Statistical Analysis

Data were shown as means ± standard errors of the mean (SME) and analyzed using one-way analysis of variance (ANOVA) with the LSD post hoc test using SPSS 23.0 (IBM, Armonk, NY, USA). Two-sided *P* values < 0.05 were defined as statistically significant. GraphPad Prism 8.0 (GraphPad Software Inc., San Diego, CA, USA) was also used for the statistical analysis of the histograms.

## 3. Results

### 3.1. RIP3 And MLKL Phosphorylation Increased with Time after IVC Thrombosis

**Figure 1:**
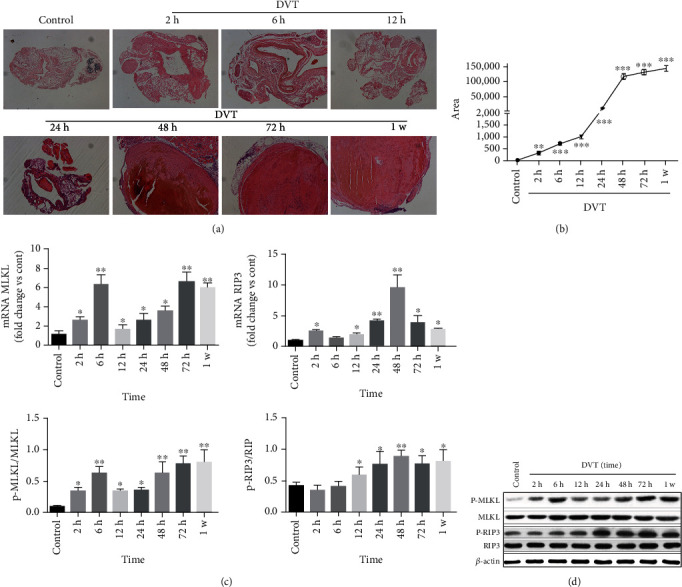
RIP3 and MLKL phosphorylation increased with time after IVC thrombosis. RIP3 and MLKL phosphorylation increased with time after IVC thrombosis. (a) H&E staining to observe thrombosis (*n* = 6, scale = 50 *μ*m, 100x). (b) Cross-sectional thrombus area measured on H&E images by Image-Pro Plus 6.0. (c) The expression level of MLKL mRNA and RIP3 mRNA in normal IVC wall and IVC wall of different time points after IVC ligation were determined by qRT-PCR. GAPDH was used as an internal reference. (d) Western blot for the detection of RIP3, p-RIP3, MLKL, and p-MLKL proteins in the IVC wall, using *β*-actin as the internal reference. The ImageJ image software analysis was used to test and analyze the expression ratios. The bar chart shows the ratio of protein gray value to total protein gray value. Six mice were tested at each time point. ANOVA was used for statistical analysis. ^∗^*P* < 0.05, ^∗∗^*P* < 0.01, and ^∗∗∗^*P* < 0.001.

**Figure 2:**
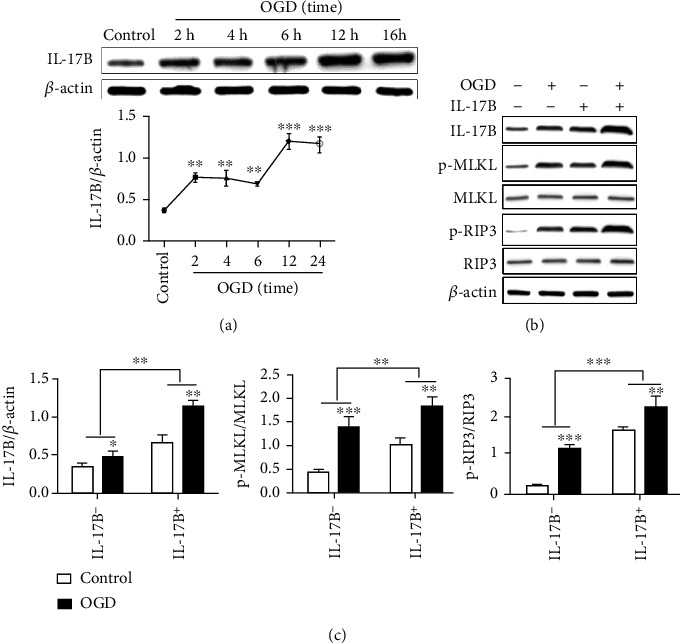
IL-17B is highly expressed in OGD cells and promotes the activation of the RIP3/MLKL pathway. IL-17B is highly expressed in OGD cells and promotes the activation of the RIP3/MLKL pathway. (a) Western blot for the detection of IL-17B protein in the OGD cells using *β*-actin as the internal reference. The line chart showed the expression trend of IL-17B protein at different OGD time. (b) Western blot for the detection of RIP3, p-RIP3, MLKL, and p-MLKL proteins in each cell group, using *β*-actin as the internal reference. The ImageJ image software analysis was used to test and analyze the expression ratios. (c) The bar chart shows the ratio of protein gray value to total protein gray value. ANOVA was used for statistical analysis. ^∗^*P* < 0.05, ^∗∗^*P* < 0.01, and ^∗∗∗^*P* < 0.001.

With the prolongation of the stenosis time, the cross-sectional area of thrombus increases, that is, the longer the stenosis time, the more serious the thrombosis (Figures 1(a) and 1(b)). Phosphorylated MLKL and RIP3 levels were low in the IVC tissue of normal WT mice. By monitoring trends in the variations in RIP3 and MLKL protein expression and phosphorylation levels of RIP3 and MLKL in the vessel wall during the process of DVT, we discovered that the increased expression of RIP3 protein and its phosphorylation level was most pronounced at 48 h after ligation, and the p-MLKL/MLKL was also increased at the same time and peaked at 72 h after ligation (Figure 1(d)). RIP3 mRNA and MLKL mRNA showed the same trend (Figure 1(c)). Simultaneously, the thrombus occupied the entire venous lumen at 48 h after ligation (Figures 2(d) and 2(e)). Thus, 48 h after ligation was used as the time point for the subsequent experiments.

### 3.2. The Activated RIP3/MLKL Pathway Is Associated with Increased IL-17B

**Figure 3:**
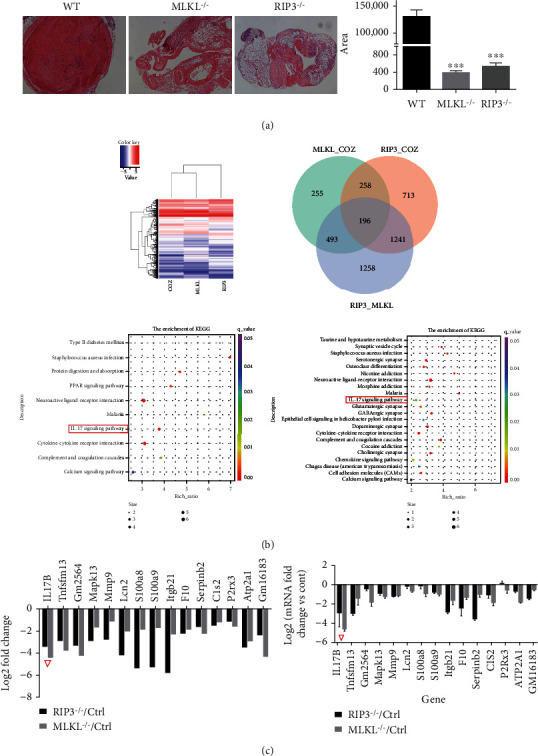
The activated RIP3/MLKL pathway is associated with increased IL-17B. The activated RIP3/MLKL pathway is associated with increased IL-17B. (a) H&E staining to observe thrombosis (*n* = 3, *scale* = 50 *μ*m, 100x). Cross-sectional thrombus area measured on H&E images by Image-Pro Plus 6.0. (b) The IVC tissues of DVT RIP3-/- mice, MLKL-/- mice and WT mice were sequenced using an Illumina platform. The sequencing strategy was PE150. The enriched KEGG items were analyzed between the DVT and control groups. The size of the dots represents the number of genes. The color of the dot represents the *P* value. (c) Q-PCR was used to verify the difference RNA with consistent changes in KEGG enrichment analysis. IL-17B was most significantly down-regulated in RIP3-/- mice and MLKL-/- mice compared with the WT mice (marked by a red triangle).

To understand the possible mechanism of endothelial injury during thrombosis, the IVC tissues of DVT RIP3-/- mice, MLKL-/- mice, and WT mice were sequenced using the Illumina sequencing platform. Before sequencing, we had a rudimentary knowledge of the thrombosis in each group of mice. Thrombosis was significantly reduced in RIP3-/- and MLKL-/- mice compared with WT mice (all *P* < 0.001) (Figure 3(a)). Among the genes in the volcano map, 1188 genes were upregulated, and 1220 genes were downregulated in the RIP3-/- mice relative to the WT mice. Meanwhile, 608 genes were upregulated, and 594 genes were downregulated in the MLKL-/- mice relative to the WT mice. Among them, the levels of RIP3, MLKL, and IL-17B were significantly increased (Figure 3(b)). The KEGG pathway analyses indicated that the interaction between cytokines and cytokine receptors, IL-17 signaling pathway, complement and coagulation cascade, calcium signaling pathway, and other aspects are significantly enriched (Figure 3(b)). Q-PCR was used to verify the difference RNA with consistent changes in KEGG enrichment analysis between the RIP3-/-/WT group and MLKL-/-/WT group. The results showed that IL-17B was most significantly downregulated in RIP3-/- mice and MLKL-/- mice compared with the WT mice (Figure 3(c)).

### 3.3. IL-17B Is Highly Expressed in OGD Cells and Promotes the Activation of the RIP3/MLKL Pathway

Compared with the control group, the expression of IL-17B protein in the OGD group was increased (*P* < 0.05), for a short time of 2 to 6 hours, the high expression did not show a time-dependence, and the expression of IL-17B was further increased after 12 hours (Figure 2(a)). Compared with the control group, the IL-17B protein expression and the level of RIP3 and MLKL phosphorylation in the OGD group increased high (*P* < 0.05). The level of RIP3 and MLKL phosphorylation raised up more significantly after IL-17B protein intervention based on OGD (*P* < 0.05) (Figures 2(b) and 2(c)).

### 3.4. RIP3 And MLKL Were Enhanced by IL-17B and Decreased by IL-17B Antibody in Thrombogenic IVC Tissues

**Figure 4:**
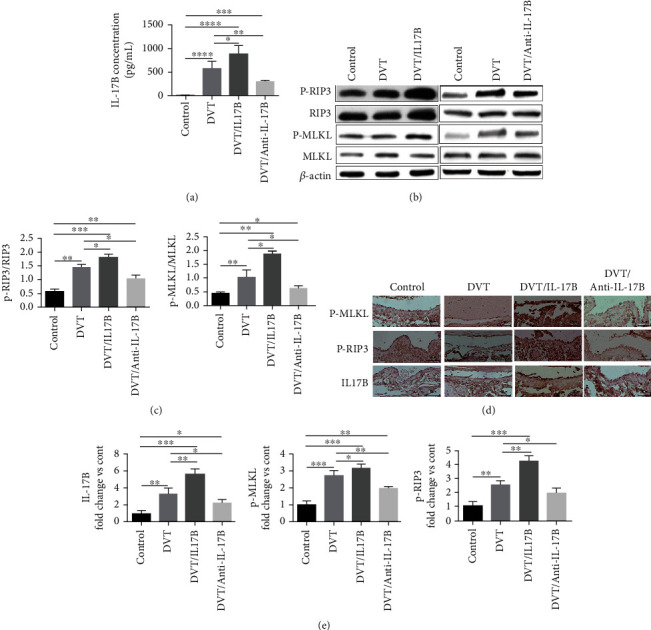
RIP3 and MLKL were enhanced by IL-17B and decreased by IL-17B antibody in thrombogenic IVC tissues. (a) ELISA was used to detect the expression of IL-17B in the IVC wall in each group. (b) Western blot of RIP3, p-RIP3, MLKL, and p-MLKL proteins in the IVC wall, using *β*-actin as the internal reference. (c) ImageJ image software analysis was used to determine the p-RIP3/RIP3 and p-MLKL/MLKL ratios in the IVC wall in each group. The bar charts show the ratio of protein gray value to total protein gray value. (d) Immunohistochemistry was used to determine the expression of p-MLKL, p-RIP3, and IL-17B proteins in each group (*n* = 6, scale = 50 *μ*m, 200x). The positive expression of the protein is shown as sepia particles. (e) The positive expression of the proteins was observed by an optical microscope. Image-Pro Plus 6.0 software was used to calculate the positive rate. The bar charts show the ratio of positive expression of the protein value to the control group. Six mice were tested in each group. ANOVA was used for statistical analysis. ^∗^*P* < 0.05, ^∗∗^*P* < 0.01, and ^∗∗∗^*P* < 0.001.

IL-17B levels increased with DVT, and L-17B levels were decreased by the anti-IL-17B antibody (Figure 4(a)). IL-17B enhanced the expression of RIP3 and MLKL in the IVC wall of DVT mice, as well as their phosphorylation levels (Figures 4(b) and 4(c)), thereby indicating necroptosis induction. The effect of IL-17B on necroptosis was further confirmed by the recovery experiment. With IL-17B antibody treatment, the p-RIP3/RIP3 ratio and the p-MLKL/MLKL ratio were reduced in WT mice (Figures 4(b) and 4(c)). These findings were confirmed by the immunohistochemistry analysis that showed that IL-17B, p-MLKL, and p-RIP3 were increased with DVT and IL-17B treatment but decreased with anti-IL-17B (Figures 4(d) and 4(e)).

### 3.5. IL-17B Accelerates Vascular Injury by Increasing the Expressions of RIP3 and MLKL and Their Phosphorylation

**Figure 5:**
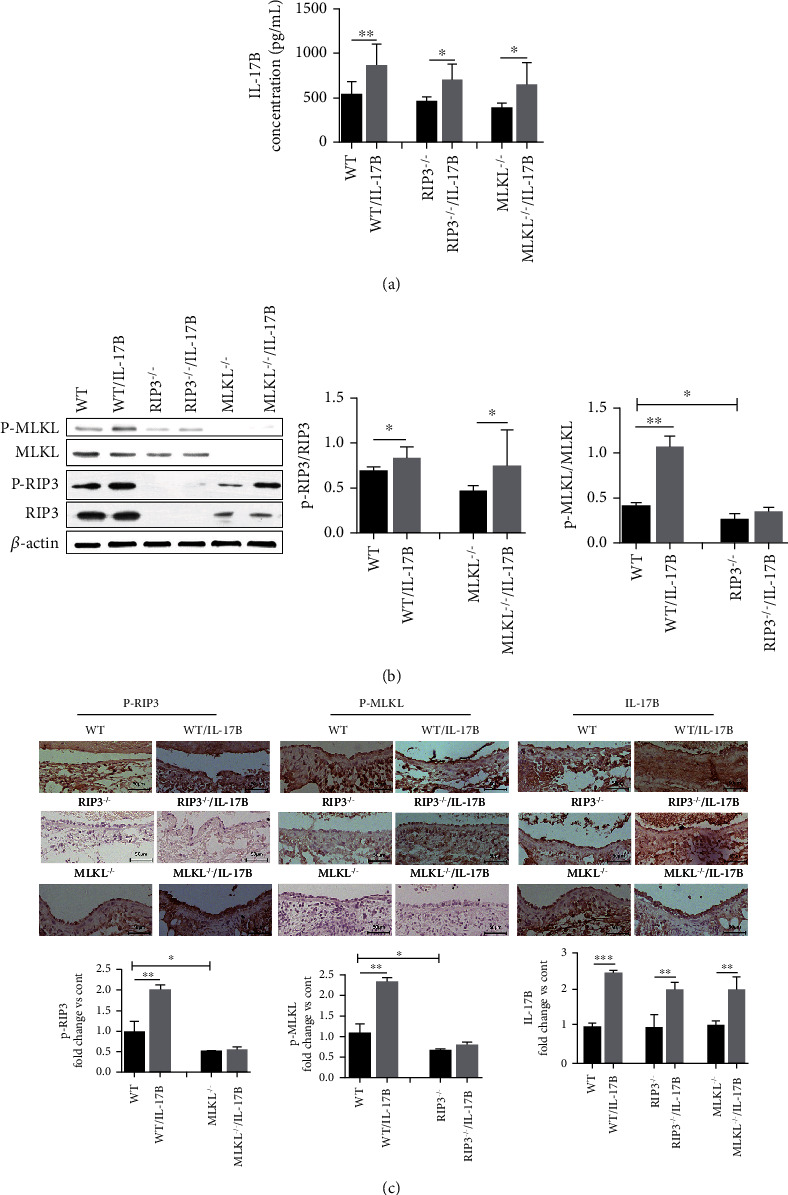
IL-17B accelerates vascular injury by increasing the expressions of RIP3 and MLKL and their phosphorylation. (a) ELISA was used to test the expression of IL-17B in the thrombogenic IVC wall of the WT and knockout mice, and with or without IL-17B interference. (b) Protein expression of RIP3, p-RIP3, MLKL, and p-MLKL in the IVC wall in each group by western blot, with the internal reference of *β*-actin as the control. The ratio of protein gray value to total protein gray value was analyzed using the ImageJ image software. (c) Immunohistochemistry was used to determine the expression of the p-RIP3, p-MLKL, and IL-17B proteins in each group. The Image-Pro Plus 6.0 software was used to calculate the positive rate. The bar chart shows the ratio of positive expression of the IL-17B protein value to the control group (*n* = 6, scale = 50 *μ*m, 200x). Six mice were tested in each group. ANOVA was used for statistical analysis. ^∗^*P* < 0.05, ^∗∗^*P* < 0.01, and ^∗∗∗^*P* < 0.001.

To further elucidate the effect of IL-17B on venous injury and thrombus formation, RIP3-/- mice and MLKL-/- mice were used. IL-17B was upregulated and positively correlated with RIP3 and MLKL in the thrombogenic IVC wall of WT mice (Figure 5(a)). As RIP3 is an upstream factor of MLKL, RIP3 knockdown led to a significant reduction in MLKL expression compared with WT mice (*P* < 0.05). There were no differences in the expression of MLKL in RIP3-/- mice with and without IL-17B treatment (*P* > 0.05). The p-RIP3/RIP3 ratio in MLKL**-/-** mice was decreased (*P* < 0.05), but that ratio increased in MLKL**-/-** mice with IL-17B treatment (*P* < 0.05, relative to MLKL**-/-** mice without IL-17B treatment) (Figures 5(b) and 5(c)). These results suggest that IL-17B accelerates vascular injury mainly by facilitating the expression of RIP3 and MLKL and their phosphorylation.

### 3.6. IL-17B Promotes the Expression of Inflammatory Factors Mainly through the Activation of RIP3/MLKL Pathway

**Figure 6:**
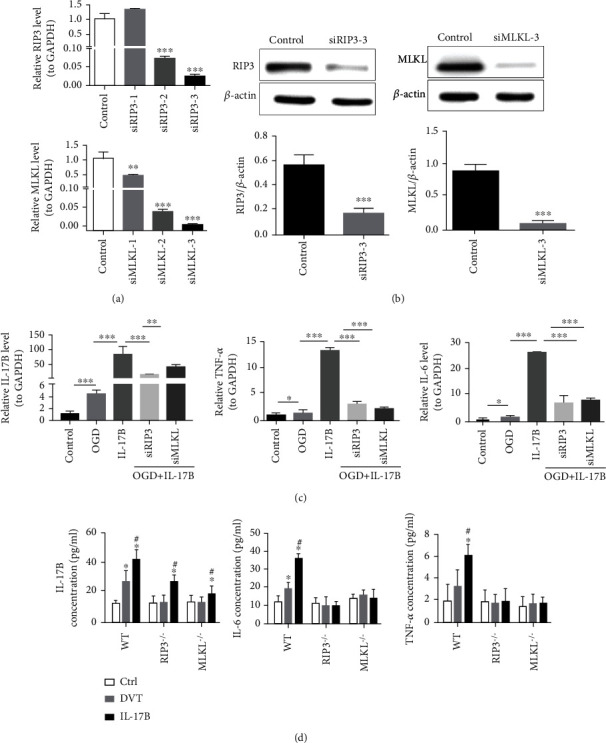
Il-17B promotes the expression of inflammatory factors mainly through the RIP3/MLKL pathway. (a) qRT-PCR was used to detect the interference efficiency of different siRIP3 and siMLKL. GAPDH was used as an internal reference. (b)Western blot was used to detect the expression of RIP3 and MLKL protein in siRIP3, siMLKL, and normal cells, with *β*-actin as an internal reference. (c) The relative expression of IL-17B, IL-6, and TNF-*α* mRNA in each cell group were detected by qRT-PCR. GAPDH was used as an internal reference. (d) ELISA was used to test the plasma, TNF-*α*, and IL-6 in each mice group. Six mice were tested in each group. ANOVA was used for statistical analysis. ^∗^*P* < 0.05, ^∗∗^*P* < 0.01, and ^∗∗∗^*P* < 0.001.

To determine the potential effects of IL-17B, RIP3, and MLKL on the inflammation of the vascular endothelial cell, we silenced RIP3 and MLKL in ECV304 cells. The RIP3 and MLKL knockdown efficiencies in ECV304 cells were evaluated by Western blot, which showed effective silencing (Figures 6(a) and 6(b)). Compared with the control group, the relative expression of IL-17B, IL-6, and TNF-*α* mRNA increased after the establishment of OGD model (all *P* < 0.05). The relative expression levels of IL-6 and TNF-*α* mRNA were further increased after added IL-17B protein on the basis of OGD. After transfection with siRIP3 or siMLKL, the relative expression of IL-17B, interleukin 6 (IL-6), and tumor necrosis factor *α* (TNF-*α*) mRNA was downregulated even after the intervention of IL-17B in OGD cells (*P* < 0.001) (Figure 6(c)). These results suggested that OGD promoted the expression of IL-17B, IL-6, and TNF-*α* mRNA in ECV304 cells. IL-17B aggravated the expression of IL-6 and TNF-*α* mRNA in ECV304 cells after OGD injury. After interfering with RIP3 and MLKL, the expression of IL-6 and TNF-*α* mRNA was inhibited, and the expression of IL-17B mRNA was also adversely affected.

Similarly, in vivo, the plasma IL-17B, IL-6, and TNF-*α* were significantly increased after thrombosis in WT mice, and the plasma IL-6 and TNF-*α* was increased more significantly after the intervention of IL-17B protein on the basis of ligation of IVC (all *P* < 0.05). But, in RIP3-/- mice or in MLKL-/- mice, IL-17B had little effect on the plasma IL-6 and TNF-*α* (all *P* > 0.05) (Figure 6(d)). These results further suggest that IL-17B promotes the expression of inflammatory factors by activating the RIP3/MLKL signal pathway.

### 3.7. Necroptosis of the Vessel Wall Facilitated by IL-17B Aggravates Thrombosis

**Figure 7:**
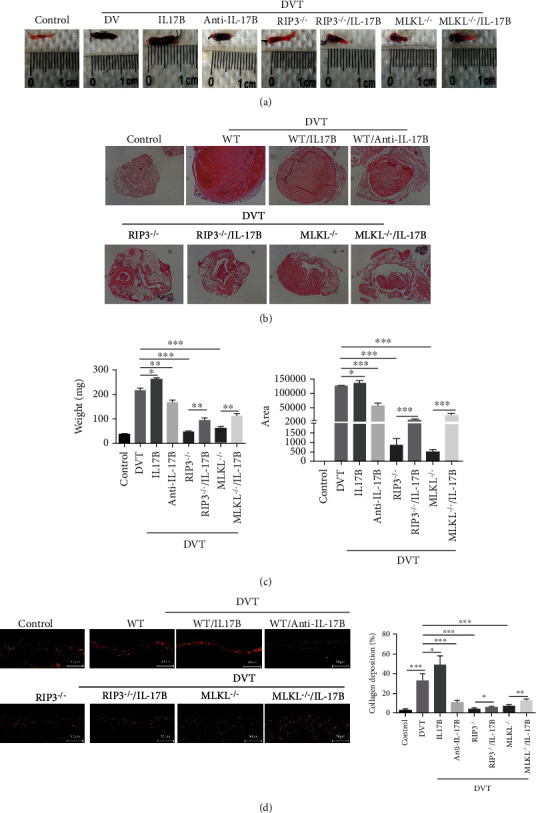
Necroptosis of the vessel wall facilitated by IL-17B aggravates thrombosis. (a) Gross specimens of IVC tissues (containing the thrombus) of each group were obtained from the ligation site to the bifurcation of the iliac vein and drained on absorbent paper. (b) HE staining of the thrombus in each group under a 40x light microscope. (c) The weight of the thrombi was measured by electronic balance. The cross-sectional thrombus area was measured on HE images using Image-Pro Plus 6.0. (d) Sirius red staining was done on the vein wall injury conditions. Observation with lab.A1 microscope (200x), and red represents the collagen, green represents the nucleus, and the rest is yellow. Five visual fields were randomly selected for double-blind observation. Collagen content was calculated using Image-Pro Plus 6.0 software. ANOVA was used for statistical analysis. ^∗^*P* < 0.05, ^∗∗^*P* < 0.01, and ^∗∗∗^*P* < 0.001.

We explored the role of IL-17B in vascular endothelial necroptosis during DVT, with the ultimate goal of understanding their roles in regulating thrombosis. The results showed that the weight and cross-sectional area of the thrombus were increased by IL-17B and decreased by the IL-17B antibody in WT mice. Compared with the DVT group, the weight of the thrombosis in DVT/IL-17B was increased (*P* < 0.05), and so was the cross-sectional area of the thrombus (*P* < 0.05). With anti-IL-17B treatment, the weight of the thrombus was reduced significantly compared with the DVT group (*P* < 0.01), and so was the cross-sectional area (*P* < 0.01). Furthermore, the weight of the thrombus was remarkably decreased in RIP3-/- mice and in MLKL**-/-** mice with or without IL-17B treatment, especially in RIP3-/- mice (*P* < 0.001) (Figures 7(a) and 7(c)). These findings suggest that the necroptosis pathway was enhanced by IL-17B, thus promoting thrombosis.

To further investigate the matrix changes, Sirius red staining was done on the vein wall injury conditions. With or without IL-17B treatment, the fiberpolarity was significantly altered in WT mice with thrombosis, suggesting collagenolysis. In RIP3-/- mice and in MLKL**-/-** mice, collagenolysis was increased, but not obvious (Figure 7(d)). It can be inferred that IL-17B aggravating the damage of vein wall, and IL-17B antibody inhibiting RIP3/MLK pathway or knocking out RIP3 or MLK genes can play a protective role in the damage of the vascular wall caused by acute DVT.

## 4. Discussion

The pathophysiological mechanisms of vascular endothelial injury during DVT are poorly understood, especially the role of necroptosis and IL-17B. Therefore, this study investigated whether vascular endothelial necroptosis is involved in DVT and how IL-17B facilitates necroptosis signaling. The results suggested that necroptosis plays a crucial role in vascular injury during thrombosis formation, and IL-17B could enhance the necroptosis pathway. This study may help identify potential prevention or therapeutic targets to prevent or mitigate the complications of DVT.

Venous stasis-induced thrombosis is a well-established model for mimicking human DVT [35]. In the present study, we established a DVT mouse model by stenosis of the IVC. Thrombus formation began at 2 h after ligation of the IVC, peaked 48 h later, and lasted the 7-day study period. The results are similar to those of previous studies that showed that thrombi start as early as 3-5 h after IVC ligation [38, 39], grow over the course of a week, and persist for at least 2 weeks [8]. Thrombosis inevitably leads to vascular endothelial injury, which in turn, aggravates thrombosis. RIP3 and MLKL are involved in necroptosis and represent injury in tissues or cells [25–27, 29, 40] and were found at low levels in the IVC tissues of WT mice in this study; but with the formation of thrombi, they were significantly upregulated, along with IL-17B. Moreover, their increased expression was consistent with the trend of thrombosis. While IL-17B is expressed in various tissues at low amounts [30, 41, 42], we found that IL-17B was also expressed in the IVC tissues of normal WT mice at a very low level and increased in thrombogenic IVC tissue. The KEGG enrichment analyses indicated that the highly expressed IL-17B in the vascular wall during thrombosis was significantly associated with the RIP3/MLKL signaling pathway.

Venous blood is the only source of oxygen supply to the venous wall. The decrease of new blood supply after thrombosis leads to local hypoxia of the venous wall [43], which links blood flow stagnation with subsequent inflammatory lesions to the vascular wall leading to potentially permanent damage that is particularly important for the development of PTS [44]. Therefore, we established a model of vascular endothelial injury after thrombosis by means of OGD. The expression of IL-17B protein in the OGD group was increased, for a short time of 2 to 6 hours, the high expression did not show a time dependence, and the expression of IL-17B was further increased after 12 hours. Meanwhile, in OGD cells, the expression levels of RIP3 and MLKL proteins were increased, and IL-17B could further promote both of them. In vivo, the IL-17B, RIP3, p-RIP3, MLKL, and p-MLKL proteins were all increased to different degrees at 48 h after IVC ligation, and the expression of MLKL peaked at 72 h after ligation. IL-17B can clearly upregulate the expression of RIP3 and MLKL and their phosphorylation levels, indicating upregulated necroptosis in the thrombogenic vascular wall. IL-17B can aggravate vascular endothelial necroptosis in the process of thrombosis.

The molecular mechanisms underlying necroptotic cell death have been well studied over the past decade. Most of the roles attributed to necroptosis have arisen from studies of RIP3**-/-** mice and MLKL**-/-** mice [25, 40, 45]. In this study, RIP3**-/-** and MLKL**-/-** mice were used to further demonstrate this mechanism. As RIP3 is an upstream factor of MLKL, RIP3 interference led to a remarkable reduction in MLKL expression, and IL-17B would not change this trend. The p-RIP3/RIP3 ratio in MLKL**-/-** mice were also decreased, but not as much as the p-MLKL/MLKL ratio in RIP3**-/-** mice. On the contrary, with IL-17B antibody treatment, the p-RIP3/RIP3 ratio and the p-MLKL/MLKL ratio were reduced in IVC tissues. Similarly, the weight and cross-sectional area of the thrombi were increased by IL-17B and decreased by IL-17B antibody in WT mice. Furthermore, thrombus formation was significantly reduced in RIP3-/- mice and in MLKL**-/-** mice, especially in RIP3-/- mice. With IL-17B treatment, thrombus formation increased slightly in knockout mice, and IL-17B had a smaller effect on thrombosis in RIP3-/- mice compared with MLKL**-/-** mice. In this study, we confirmed that RIP3 loss was more effective than MLKL loss at thrombosis, which is probably because RIP3 regulates more than just MLKL-dependent necroptosis [46]. RIP3 also promotes platelet activation and thrombus formation independently of MLKL [47]. All these findings indicated that vascular endothelial necroptosis is involved in DVT and that IL-17B could facilitate necroptosis signaling, thus promoting thrombosis, but this is not the only pathway through which IL-17B plays a role in DVT.

As mentioned earlier, research on cell necrosis is a necessary way to investigate the relationship between DVT and inflammation. Several groups have reported that IL-17B stimulates the expression of TNF-*α*, IL-1, and IL-6 in macrophages [30, 42]. In this study, we found that the relative expression of IL-17B, IL-6, and TNF-*α* mRNA increased after the establishment of OGD model, and IL-6 and TNF-*α* mRNAs were further increased after added IL-17B protein on the basis of OGD. However, after transfection with siRIP3 or siMLKL, the relative expression of IL-17B, IL-6, and TNF-*α* mRNA was downregulated even after the intervention of IL-17B in OGD cells. These results suggested that OGD promoted the expression of IL-17B, IL-6, and TNF-*α* in vascular endothelial cell cells, and IL-17B aggravated the expression of IL-6 and TNF-*α* mainly via activating the RIP3/MLKL signaling pathway. Consistent with the results of vitro experiments, the plasma IL-6 and TNF-*α* were significantly elevated in DVT WT mice, and they were further promoted by IL-17B. But, in RIP3-/- mice or in MLKL-/- mice, IL-17B had little infection to the plasma IL-6 and TNF-*α*. These results further suggest that IL-17B promotes the expression of inflammatory factors by activating the RIP3/MLKL signal pathway.

The limitation of this study is that we just showed that IL-17B could aggravate endothelial necroptosis, but the potential mechanism underlying the effect of IL-17B on vascular endothelial necroptosis should also be further explored. In addition, the relationship between IL-17B, necroptosis, and inflammatory injury is worthy of further exploration. Even so, we found that increased IL-17B increased the activation of RIP3 and MLKL and promoted DVT. Therefore, the prevention of vascular endothelial necroptosis might be an effective treatment to reduce thrombosis-associated injury, and IL-17B might be a promising therapeutic target for the protection of vascular endothelial cells during DVT.

## 5. Conclusions

Vascular endothelial necroptosis plays a crucial role in thrombosis formation. IL-17B could enhance the necroptosis pathway, as shown by the RIP3 and MLKL expressions. This study helps a better understanding of the mechanisms of DVT and could help the development of potential prevention or therapeutic targets to prevent or mitigate the complications of DVT.

## Data Availability

The data used to support the findings of this study are included within the article.
